# Comparison of Clinical Data Between Patients With Complications and Without Complications After Spinal Tuberculosis Surgery: A Propensity Score Matching Analysis

**DOI:** 10.3389/fsurg.2022.815303

**Published:** 2022-03-29

**Authors:** Liyi Chen, Chong Liu, Zhen Ye, Wuhua Chen, Xuhua Sun, Jiarui Chen, Hao Li, Tuo Liang, Shengsheng Huang, Jie Jiang, Tianyou Chen, Hao Guo, Yuanlin Yao, Shian Liao, Chaojie Yu, Shaofeng Wu, Binguang Fan, Xinli Zhan

**Affiliations:** First Affiliated Hospital, Guangxi Medical University, Nanning, China

**Keywords:** propensity score matching, logistic, complications, spinal tuberculosis, albumin

## Abstract

**Purpose:**

This study used a propensity score matching (PSM) analysis to explore the risk factors of post-operative complications and compared the differences in clinical data between them following spinal tuberculosis surgery.

**Methods:**

The clinical data of patients with spinal tuberculosis were collected in our hospital from June 2012 to June 2021, including general information, laboratory results, surgical information, and hospitalization costs. The data were divided into two groups: complication and without complication groups. The baseline data of the two groups were obtained using the PSM analysis. Univariate and multivariate logistic analyses were used to analyze the differences between the two groups.

**Results:**

A total of 292 patients were included in the PSM analysis: 146 patients with complications and 146 patients without complications. The operation time, incision length, hospital stay, and albumin quantity in the complications group were 162 ± 74.1, 11.2 ± 4.76, 14.7 ± 9.34, and 1.71 ± 2.82, respectively, and those in the without complication group were 138 ± 60.5, 10.2 ± 3.56, 11.7 ± 7.44, and 0.740 ± 2.44, respectively. The laboratory costs, examination costs, guardianship costs, oxygen costs, and total costs in the complications group were higher than those in the without complication group. A significant difference was observed in the albumin quantity by logistic regression analysis (*P* < 0.05).

**Conclusion:**

Several costs in the complication group were higher than in the without complication group. The albumin quantity may be an independent factor to predict post-operative complications of spinal tuberculosis by logistic regression analysis.

## Introduction

Spinal tuberculosis is the most common form of extrapulmonary tuberculosis (1). It has become a major health concern in developing countries due to the emergence of drug-resistant strains (2, 3). Surgical treatment is known to be the best solution in case the anti-tuberculosis treatment fails (4). However, surgeries are sometimes associated with certain unexpected complications. The results of Zhou et al.'s study revealed that 102 patients with L1–2 vertebral tuberculosis had 14 patients with pulmonary complications after the surgery (5). Zhuang et al. applied oblique lateral interbody fusion to lumbar spinal tuberculosis with 10 (16.95%) complications, including peritoneal injury, neurological injury, infection of incisions, segmental artery, iliac vein lacerations, and instrument failure. However, the anterior-only approach was associated with 37.5% complications (4). Similarly, Susanta et al. reported one case (2.3%) of sinus formation and two cases (4.7%) of superficial wound infection after thoracic tuberculosis surgery (6). Surgeons should completely understand the surgical approach and post-operative complications of spinal tuberculosis, which is of great significance in treating the disease (7).

The global tuberculosis situation is particularly tense in developing countries. The incidence of pulmonary or spinal tuberculosis is low in developed countries, however, the incidence is high in developing countries. The incidence of tuberculosis is high in the African region, which accounted for 71% of global tuberculosis and AIDS cases in 2018 (8). The backward economies of developing countries cannot provide good sanitation and treatment facilities, which is also the reason for the high incidence of tuberculosis (9). Insufficient course of antituberculosis therapy after spinal tuberculosis surgery due to financial burden, which may increase the recurrence of spinal surgery.

In the past, our team's research had found that there were various complications of spine surgery. There was a potential risk of cerebrospinal fluid leakage and reduced albumin after surgery for spinal tuberculosis (10, 11). In addition, we also found a risk of internal fixation failure following spinal surgery (12). In a recent study of spinal tuberculosis, our team found that spinal tuberculosis was prone to recurrence after surgery and the risk of pulmonary embolism after multiple surgical treatments (13). Therefore, we collected a large number of clinical data on spinal tuberculosis surgery with the aim of predicting the risk of complications of spinal tuberculosis.

Although PSM analysis has been favored in spinal surgery, including cervical spine surgery (14), thoracic surgery (15), and lumbar spine surgery (16), only a few reports are available on spinal tuberculosis with PSM analysis. However, a combination of PSM analysis and logistic regression analysis for spinal tuberculosis analysis has not been reported. This study was the first to apply a combination of PSM analysis and logistic regression analysis to study the complications after spinal tuberculosis. We compared the clinical data of complication and without complication groups and explored the factors related to complications after tuberculosis surgery.

## Materials and Methods

**Figure 1:**
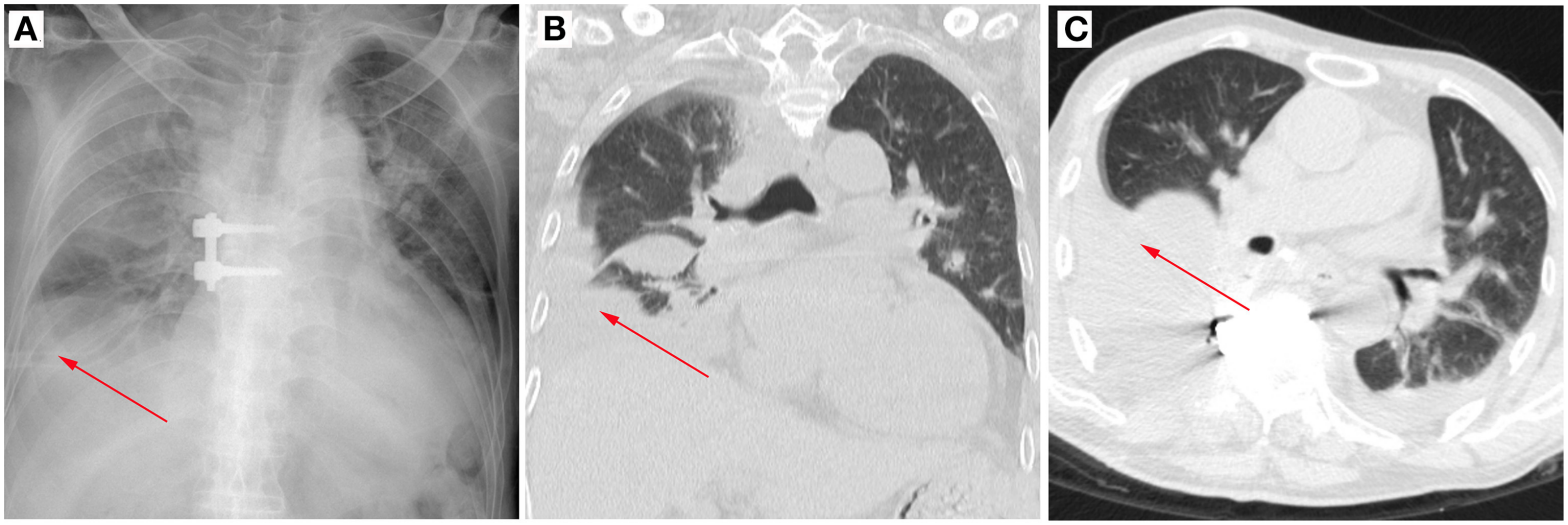
A typical case of post-operative pleural effusion after spinal tuberculosis. The arrow marks the location of the pleural effusion. **(A)** Post-operative X-ray image in the posterior–anterior position. **(B)** Post-operative CT image in the coronal position. **(C)** Post-operative CT image in the cross-section.

**Figure 2:**
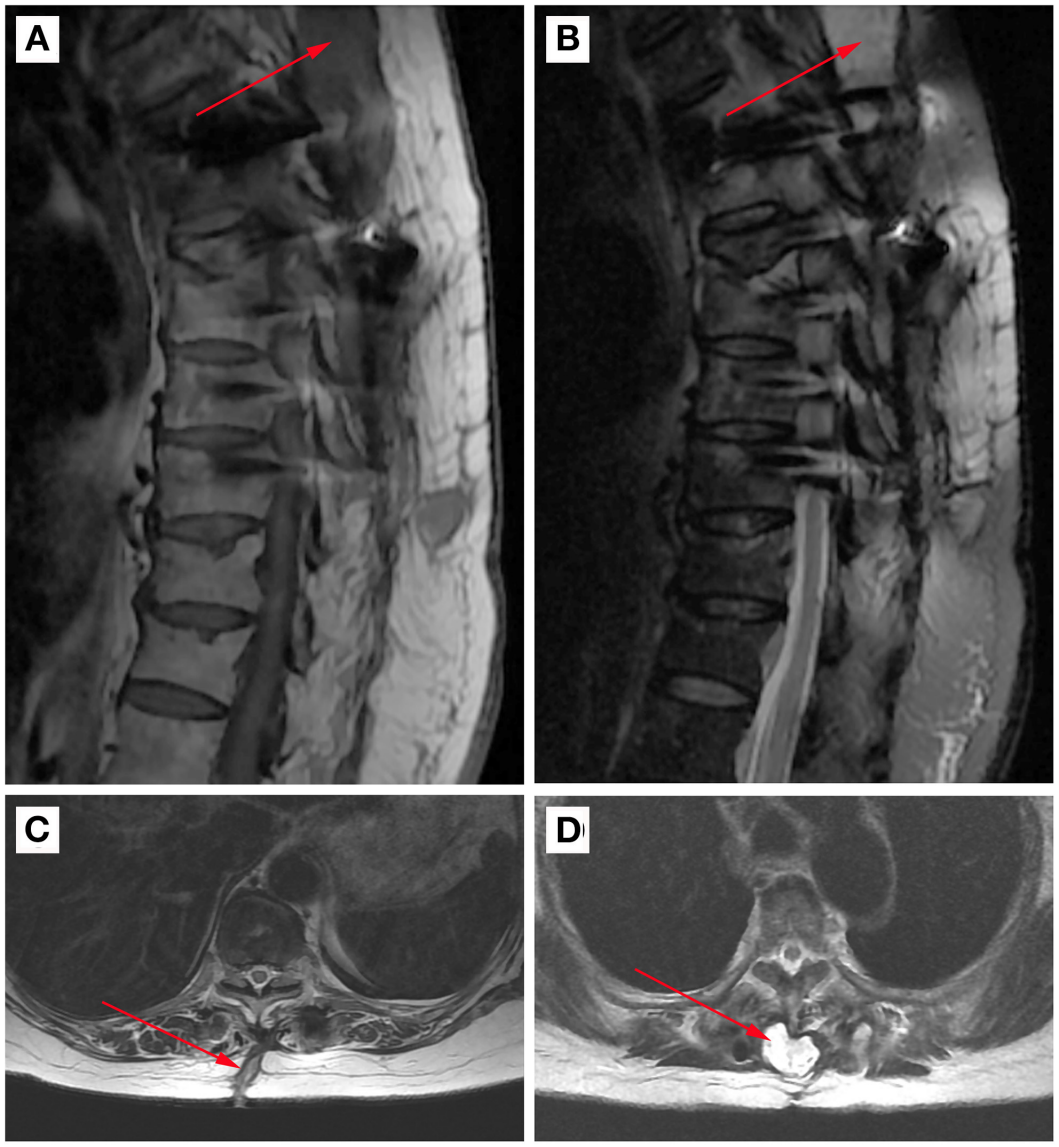
A typical case of post-operative wound infection of spinal tuberculosis. The arrow marks the location of inflammatory pus. **(A)** Post-operative MRI image of the sagittal position T1 sequence. **(B)** Post-operative MRI image of the sagittal position T2 sequence. **(C)** Post-operative MRI image of the cross-section T1 sequence. **(D)** Post-operative MRI image of the cross-section T2 sequence.

**Figure 3:**
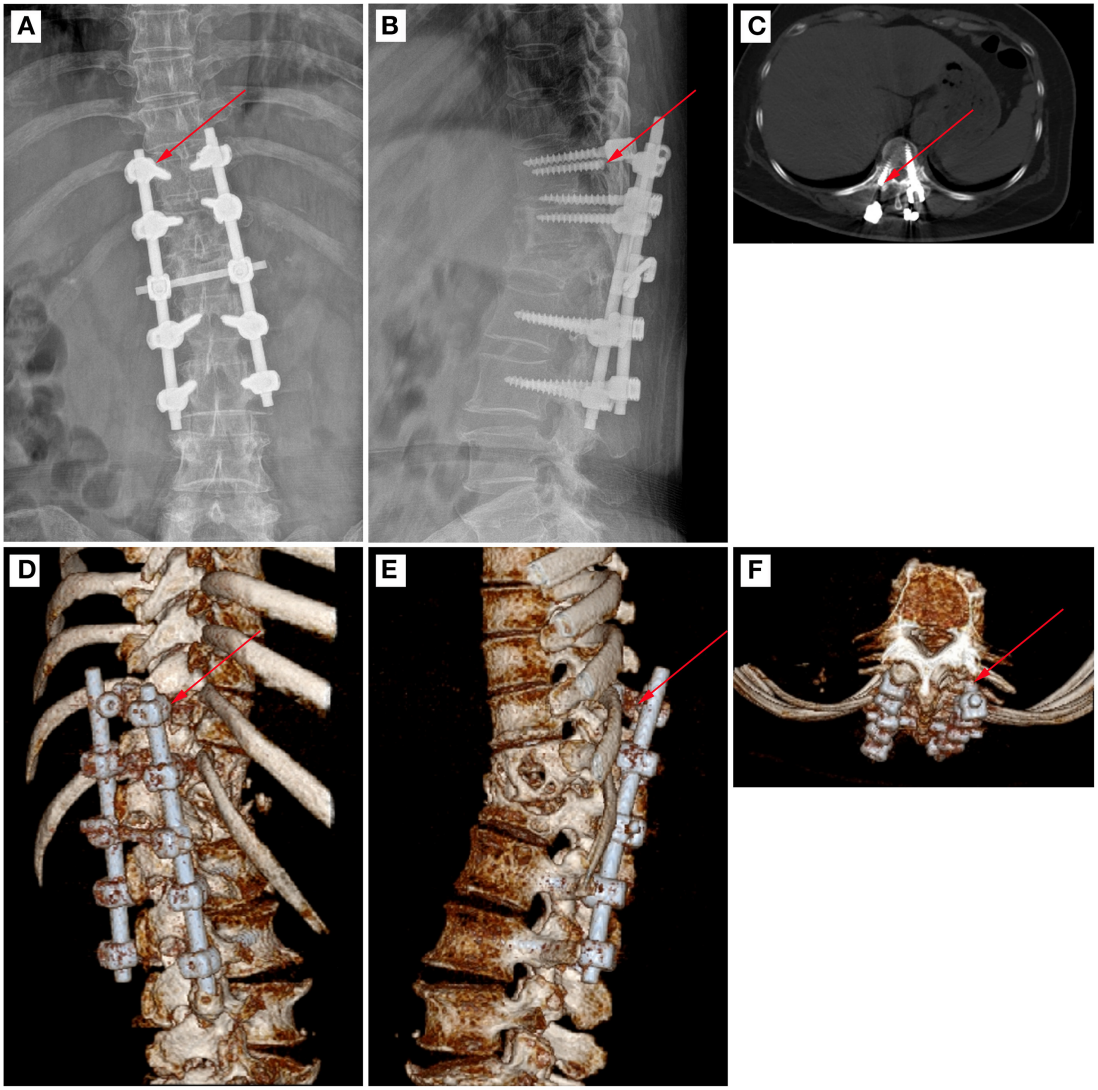
A typical case of pedicle screw fracture after spinal tuberculosis surgery. The arrow marks the location of pedicle screw fracture. **(A)** Post-operative X-ray image in the posterior–anterior position. **(B)** Post-operative X-ray image in the lateral position. **(C)** Post-operative CT image in the cross-section. **(D)** Post-operative CT reconstruction image in the coronal position. **(E)** Post-operative CT reconstruction image in the sagittal position. **(F)** Post-operative CT reconstruction image in the cross-section.

The clinical data of patients with spinal tuberculosis were collected in our hospital from June 2012 to June 2021, including general information, laboratory results, surgical information, post-operative complications, and hospitalization costs. A total of 495 patients were enrolled in the study, including 298 males and 197 females. All data were divided into two groups: complication group with 146 patients (Figures 1–3) and without complication group with 349 patients. In this study, 292 patients were included in the PSM analysis: 146 patients in the complication group and 146 patients in the without complication group. The criteria for inclusion were as follows: (1) without other diseases that affected post-operative recovery except hypertension and diabetes, (2) diagnosed with spine tuberculosis by pathological examination, (3) without a history of surgery affecting the spine, (4) complete clinical data, and (5) Lesions involving any part of the spine, including cervical, thoracic, lumbar, and sacral. The criteria for exclusion were as follows: (1) other diseases that affected post-operative recovery except for hypertension and diabetes, (2) without diagnosis of spinal tuberculosis by pathological examination, (3) with a history of surgery affecting the spine, and (4) incomplete clinical data. Post-operative complications of spinal tuberculosis were defined as post-operative wound infection or systemic infection, failure of internal fixation, recurrence of tuberculosis, and surgery-related diseases such as pulmonary embolism, cerebral infarction, and myocardial infarction. According to the fixed vertebral body at the upper end and the fixed vertebral body at the lower end, we choose the appropriate length of incision. “Bottle” was defined as the unit of measurement for albumin quantity. Each bottle of albumin injection was 50 ml, of which albumin accounted for 20%. This study was approved by the Ethics Committee of the First Affiliated Hospital of Guangxi Medical University.

### Data Collection

The general information of patients who underwent spinal tuberculosis surgery in our hospital was collected, including gender, body mass index (BMI), age, hypertension, diabetes, lesion segment, medical history, marriage, systolic blood pressure, diastolic blood pressure, pain, lower limb pain, number of lower limb pain, fatigue, fever, night sweats, appetite, weight loss, Oswestry Disability Index (ODI), Japanese Orthopedic Association (JOA) scores, visual analog scale (VAS), American Spinal Injury Association (ASIA) impairment scale, occupation, race, days before surgery, smoking, and drinking. The laboratory results of patients were collected, including blood glucose, blood type, C-reactive protein (CRP), hepatitis B surface antigen, white blood cells, hemoglobin, platelets, percentage of neutrophils, percentage of lymphocytes, absolute monocytes, percentage of monocytes, total bilirubin, direct bilirubin, indirect bilirubin, total protein, albumin, aspartate aminotransferase (AST), alanine aminotransferase (ALT), AST/ALT, blood urea, blood creatinine, blood uric acid, and erythrocyte sedimentation rate. The surgical information of patients was collected, including titanium cage, internal fixation, abscess, albumin quantity, recurrence, surgical approach, hospital stay, transfusion, local streptomycin, operation time, bleeding volume, red blood cell transfusion, drainage, kyphosis, post-operative complications, and incision length. The hospitalization costs of patients were collected, including antibacterial costs, western medicine costs, surgical costs, anesthesia costs, laboratory costs, examination costs, guardianship costs, transfusion costs, oxygen costs, high consumable costs, care costs, consultation fees, and total costs. Antibacterial costs were defined as the cost of treatment with antibiotics such as penicillin, streptomycin and cefuroxime etc. Western medicine costs were defined as the cost of oral medication such as painkillers, omeprazole and glutathione etc. Surgical costs were defined as the cost of lesion removal, bone graft fusion, reconstruction of spinal stability, etc., but did not include the cost of purchasing internal fixation devices. High consumable costs were defined as the purchase of internal fixation devices such as pedicle screws, titanium cages. Anesthesia costs were defined as the cost of anesthesia during surgery. Laboratory costs were defined as the cost of blood tests before and after surgery. Examine costs were defined as the cost of pre-operative and post-operative imaging examinations, such as X-ray, CT, MRI, etc. Guardianship costs were defined as the cost of monitoring vital signs after surgery. Transfusion costs were defined as the cost of blood transfusions during hospitalization. Oxygen costs were defined as the cost of oxygen therapy during hospitalization. Care costs were defined as the costs of nurses serving patients during hospital stays. Consultation costs were defined as the cost of asking other departments for consultation and treatment during hospitalization. Total costs were defined as the total cost of hospitalization.

### Statistical Analysis

The propensity score usually includes four methods, namely covariate adjustment using the propensity score, matching on the propensity score, stratification on the propensity score, and inverse probability of treatment weighting using the propensity score. The most popular of these in medical research is matching on the propensity score (17, 18). Khawar et al. constructed groups for propensity score matching research based on outcomes after hip fractures sustained in the hospital (19). Omar et al. constructed the non-elderly group and the elderly group after robotic ventral hernia repair for PSM research (20). Maze et al. used the PSM method to analyze the clinical data according to the presence or absence of intracranial hemorrhage (21). We referred to previous research strategies and used the PSM method to analyze the clinical data based on the presence or absence of complications after spinal tuberculosis surgery. PSM can reduce the interference factors of the outcome after matching the baseline data of treatment and control groups (22, 23). Although PSM analysis has been favored in spinal surgery, including cervical spine surgery (14), thoracic surgery (15), and lumbar spine surgery (16), only a few reports are available on spinal tuberculosis with PSM analysis.

Univariate and multivariate analyses were usually applied to the analysis of related factors in the study of spinal tuberculosis. A study by Surachai et al. used logistic regression analysis and found that signal cord change and notable Cobb angle could be used as predictive factors for the neurological deficit in spinal tuberculosis (24). Moreover, Wang et al. used logistic regression analysis and found that the independent risk factors of lower extremity motor or sensory deficits were age, worrying of sickness, location, and spinal compression in patients with spinal tuberculosis (25).

The RStudio software (version 1.4.1717) and R software (version 4.1.0) were used for data analysis. To eliminate the confounding factors that affected the results, we selected certain parameters for the PSM analysis, including gender, BMI, age, hypertension, diabetes, abscess, titanium cage, internal fixation, percentage of lymphocytes, albumin, and lesion segment. The balance of the matched data was tested using the classical method; the alpha was set to 5. In addition, the balance of the matched data was tested using the standard difference method; the alpha was set to 15. The data following the PSM analysis were included in univariate analysis. We refer to previous published articles (26, 27), and on the basis of fully considering the clinical significance and statistical significance of the parameters. Parameters with *P* <0.1 in the univariate analysis were included in the multivariate logistic regression analysis. The validity of the logistic regression model was evaluated by the receiver operating characteristic (ROC) curve. The statistical difference was considered as *P*-value <0.05.

## Results

**Figure 4:**
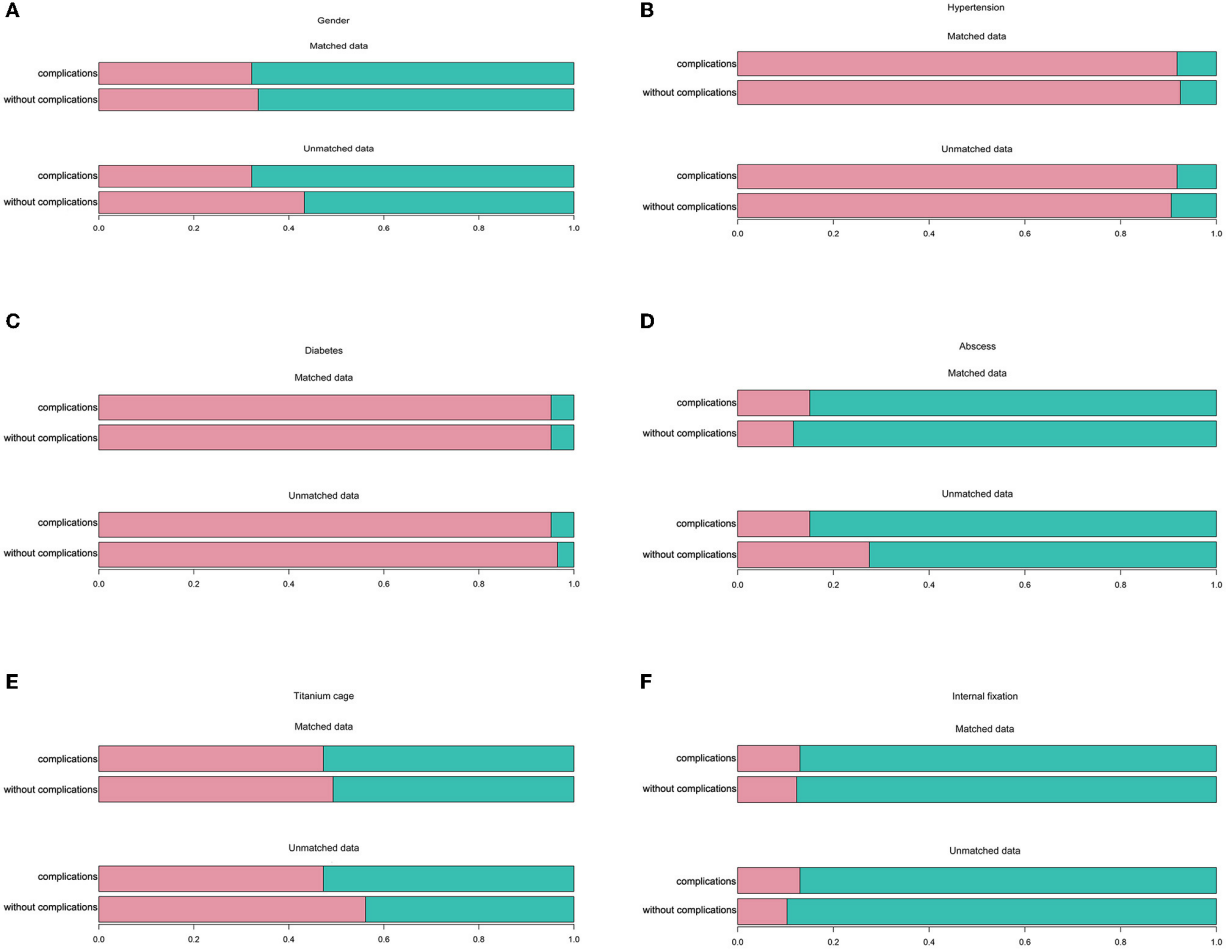
Comparison of grade clinical data between complication and without complication groups before and after propensity score matching analysis. **(A)** Gender. **(B)** Hypertension. **(C)** Diabetes. **(D)** Abscess. **(E)** Titanium cage. **(F)** Internal fixation.

**Figure 5:**
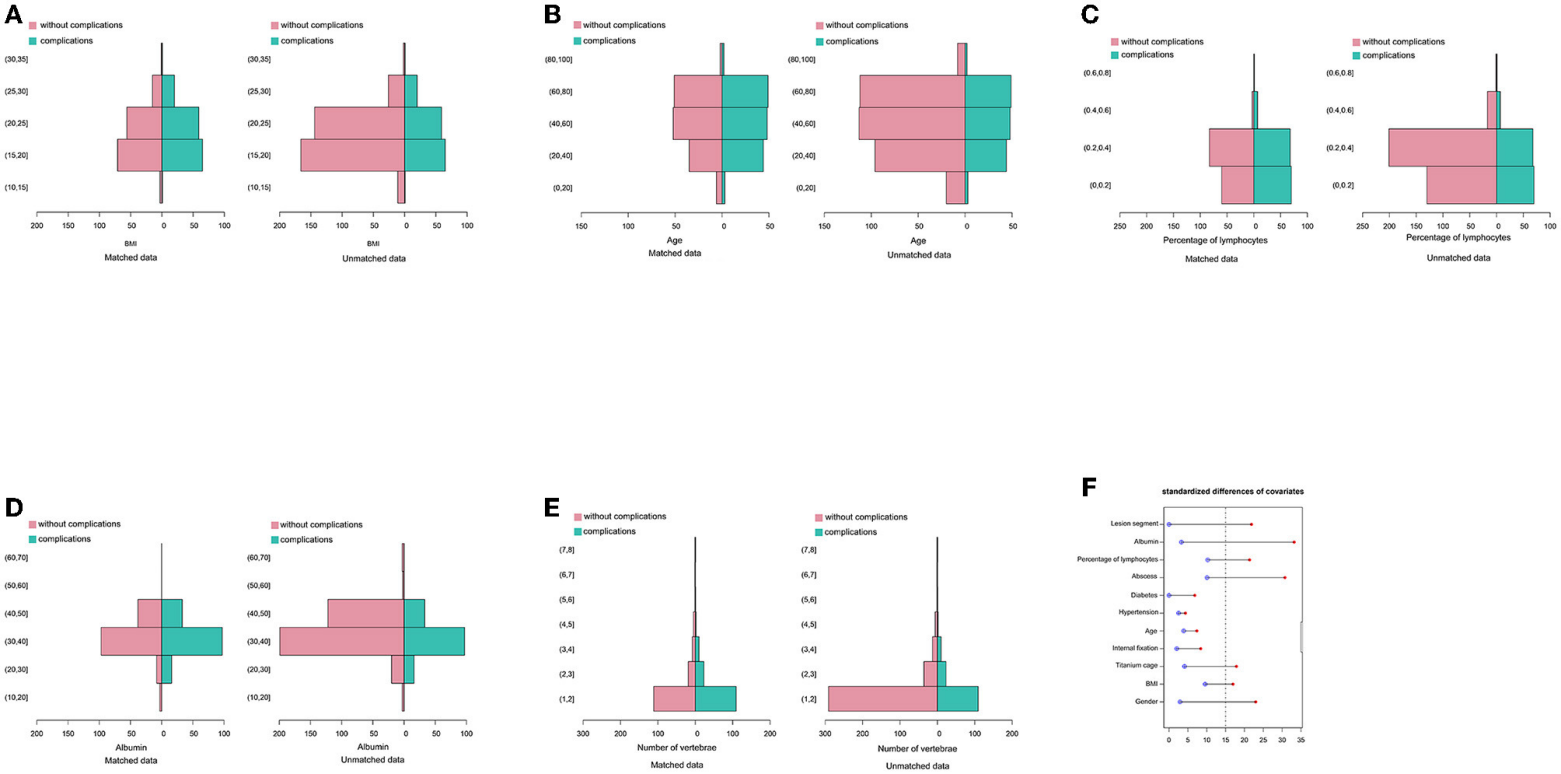
Comparison of continuous clinical data between complication and without complication groups before and after the propensity score matching analysis. **(A)** BMI. **(B)** Age. **(C)** Percentage of lymphocytes. **(D)** Albumin. **(E)** Lesion segment. **(F)** Standardized differences of covariates.

There were differences in baseline data before propensity matching, but none were statistically significant after propensity matching (Table 1). The histogram showed a difference between categorical variables, including gender (Figure 4A), hypertension (Figure 4B), diabetes (Figure 4C), abscess (Figure 4D), titanium cage (Figure 4E), and internal fixation (Figure 4F), before and after matching. The frequency distribution graph showed a difference in quantitative variables, including BMI (Figure 5A), age (Figure 5B), percentage of lymphocytes (Figure 5C), albumin (Figure 5D), and lesion segment (Figure 5E) before and after matching. The balance of the standardized differences of covariates was evaluated using the standard difference method; the results showed that the standardized difference of the matched data was significantly smaller than that of the unmatched data (Figure 5F).

**Table 1:** Baseline data before and after propensity score matching analysis.

	**Before propensity score-matching**			**After propensity score-matching**		
	**Without complications**	**Complications**	***P*** **-value**	**Without complications**	**Complications**	***P*** **-value**
	***N*** **= 349**	***N*** **= 349**		***N*** **= 349**	***N*** **= 349**	
Gender (%)						
Female	151 (43.3)	47 (32.2)	0.028^*^	49 (33.6)	47 (32.2)	0.901
Male	198 (56.7)	99 (67.8)		97 (66.4)	99 (67.8)	
Titanium cage (%)						
No	196 (56.2)	69 (47.3)	0.087	72 (49.3)	69 (47.3)	0.815
Yes	153 (48.3)	77 (52.7)		74 (50.7)	77 (52.7)	
BMI [mean (SD)]	20.25 (3.20)	20.80 (3.32)	0.084	20.49 (3.25)	20.80 (3.32)	0.414
Internal fixation (%)						
No	36 (10.3)	19 (13.0)	0.475	18 (12.3)	19 (13.0)	1
Yes	313 (89.7)	127 (87.0)		128 (87.7)	127 (87.0)	
Age [mean (SD)]	49.81 (17.89)	51.08 (16.48)	0.461	51.73 (16.54)	51.08 (16.48)	0.739
Hypertension (%)						
No	316 (90.5)	134 (91.8)	0.791	135 (92.5)	134 (91.8)	1
Yes	33 (9.5)	12 (8.2)		11 (7.5)	12 (8.2)	
Diabetes (%)						
No	337 (96.6)	139 (95.2)	0.646	139 (95.2)	139 (95.2)	1
Yes	12 (3.4)	7 (4.8)		7 (4.8)	7 (4.8)	
Abscess (%)						
No	96 (27.5)	22 (15.1)	0.004^**^	17 (11.6)	22 (15.1)	0.491
Yes	253 (72.5)	124 (84.9)		129 (88.4)	124 (84.9)	
Percentage of lymphocytes [mean (SD)]	0.24 (0.10)	0.22 (0.11)	0.028^*^	0.23 (0.09)	0.22 (0.11)	0.38
Albumin [mean (SD)]	38.43 (5.91)	36.61 (4.96)	0.001^**^	36.78 (5.45)	36.61 (4.96)	0.779
Number of vertebrae [mean (SD)]	2.17 (0.84)	2.36 (0.91)	0.024^*^	2.36 (1.07)	2.36 (0.91)	1

* *This means that the P-value is <0.05*.

** *This means that the P-value is <0.01*.

**Figure 6:**
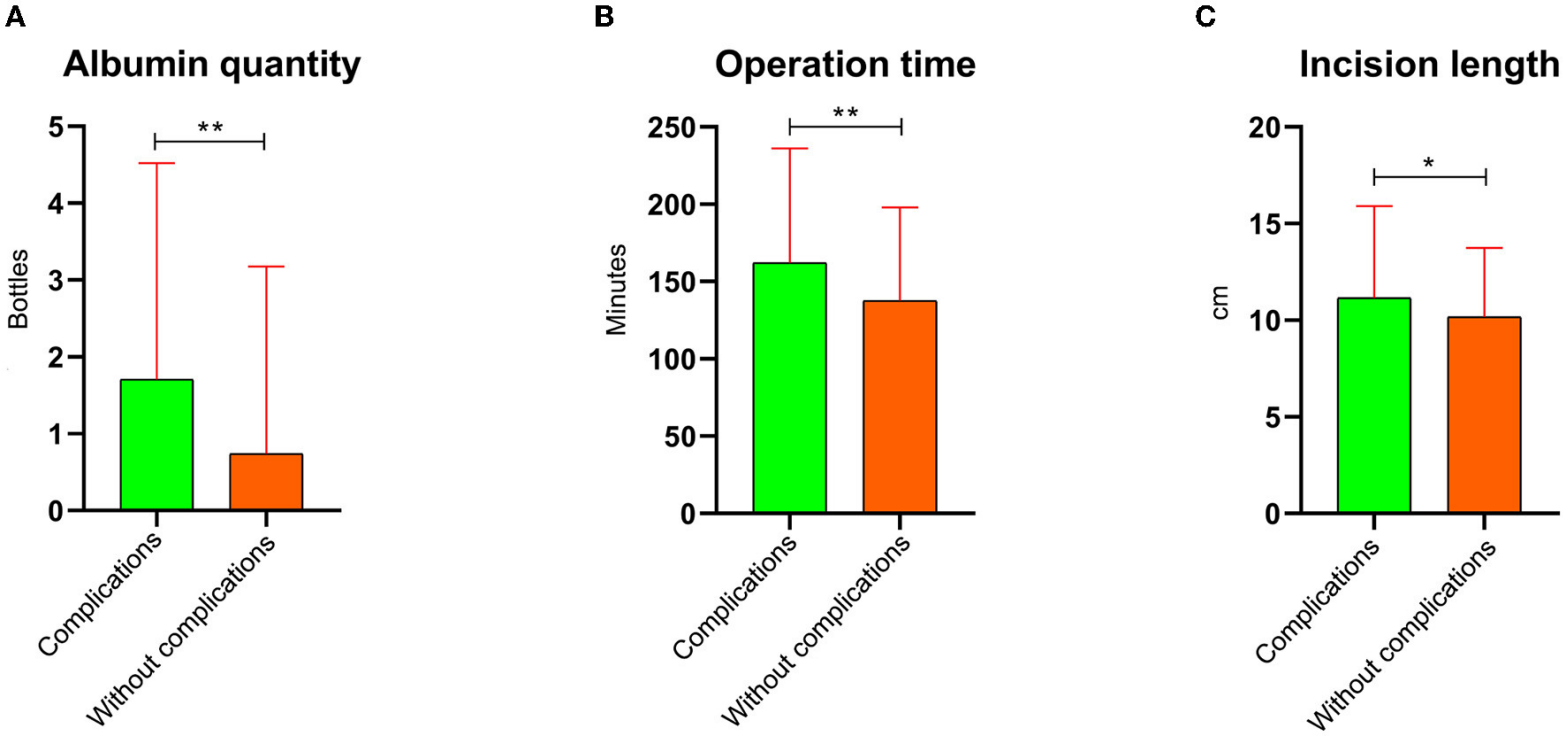
Post-operative information comparison between complication and without complication groups after the propensity score matching analysis. **(A)** Albumin quantity. **(B)** Operation time. **(C)** Incision length. *This means that the *P*-value is <0.05. **This means that the *P*-value is <0.01.

**Figure 7:**
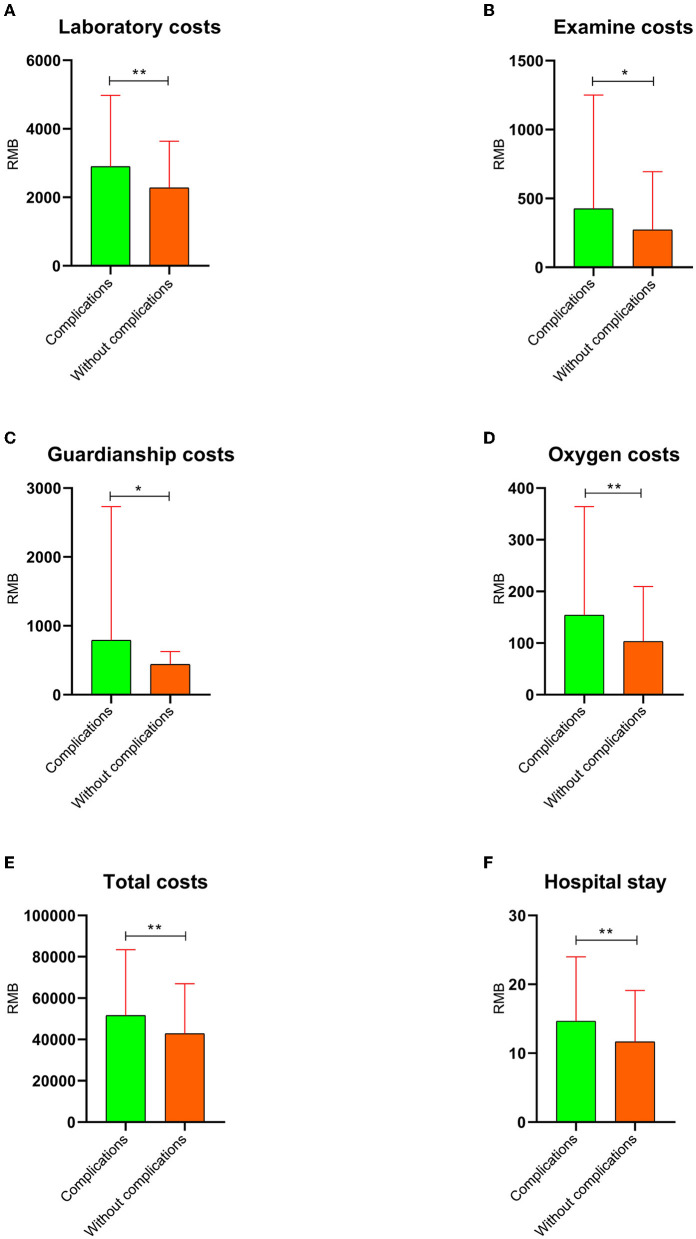
The hospital costs and days with *P* < 0.05 after the propensity score matching analysis. **(A)** Laboratory costs. **(B)** Examination costs. **(C)** Guardianship costs. **(D)** Oxygen costs. **(E)** Total costs. **(F)** Hospital stay. *This means that the *P*-value is <0.05. **This means that the *P*-value is <0.01.

The general data between the two groups using the PSM analysis revealed that the *P*-value of appetite and occupation was <0.1, and that of other parameters was more than 0.1 (Table 2). The laboratory results showed that only the comparison of AST/ALT between the two groups was <0.1 (*P* = 0.067), and there were no significant differences in other parameters (Table 3). The albumin quantity (Figure 6A), operation time (Figure 6B), incision length (Figure 6C), and hospital stay (Figure 7F) in the complication group were 1.71 ± 2.82 bottles, 162 ± 74.1 min, 11.2 ± 4.76 cm, and 14.7 ± 9.34 days, respectively, and these values in the without complication group were 0.740 ± 2.44 bottles, 138 ± 60.5 min, 10.2 ± 3.56 cm, and 11.7 ± 7.44 days, respectively (Table 4). There were statistical differences between the two groups (*P* < 0.05). In addition, there were statistical differences in the surgical approach between the two groups (*P* < 0.1). The laboratory costs (Figure 7A), examination costs (Figure 7B), guardianship costs (Figure 7C), oxygen costs (Figure 7D), and total costs (Figure 7E) in the complication group were 2,900 ± 2,080 RMB, 425 ± 826 RMB, 791 ± 1,940 RMB, 154 ± 210 RMB, and 51,700 ± 31,800 RMB, respectively, and those in the without complication group were 2,280 ± 1,360 RMB, 272 ± 422 RMB, 441 ± 186 RMB, 103 ± 106 RMB, and 42,800 ± 24,200 RMB, respectively (Table 5). These costs were higher in the complication group than in the without complication group (*P* < 0.05). There were no significant differences in other costs, including antibacterial costs, western medicine costs, surgical costs, anesthesia costs, transfusion costs, high consumable costs, care costs, and consultation costs, between the two groups (*P* > 0.05).

**Table 2:** General information comparison between complication and without complication groups after propensity score matching analysis.

	**Without complications**	**Complications**	***P*** **-value**
	**(*N* = 146)**	**(*N* = 146)**	
Gender						
Female	49 (34%)	47 (32%)	0.901
Male	97 (66%)	99 (68%)	
BMI	20.5 ± 3.25	20.8 ± 3.32	0.414
Age	51.7 ± 16.5	51.1 ± 16.5	0.739
Hypertension			
No	135 (92%)	134 (92%)	1
Yes	11 (8%)	12 (8%)	
Diabetes			
No	139 (95%)	139 (95%)	1
Yes	7 (5%)	7 (5%)	
Lesion segment	2.36 ± 1.07	2.36 ± 0.909	1
Medical history (months)	10.8 ± 32.3	7.60 ± 13.0	0.271
Marriage			
No	20 (14%)	16 (11%)	0.593
Yes	126 (86%)	130 (89%)	
Systolic blood pressure (mmHg)	127 ± 18.6	128 ± 20.3	0.659
Diastolic blood pressure (mmHg)	79.8 ± 11.6	78.7 ± 11.5	0.417
Pain			
No	7 (5%)	4 (3%)	0.539
Yes	139 (95%)	142 (97%)	
Lower limb pain			
No	66 (45%)	69 (47%)	0.814
Yes	80 (55%)	77 (53%)	
Number of lower limb pain	0.959 ± 0.931	0.897 ± 0.908	0.567
Fatigue			
No	106 (73%)	106 (73%)	1
Yes	40 (27%)	40 (27%)	
Fever			
No	102 (70%)	109 (75%)	0.433
Yes	44 (30%)	37 (25%)	
Night sweats			
No	113 (77%)	119 (82%)	0.469
Yes	33 (23%)	27 (18%)	
Appetite			
No	65 (45%)	48 (33%)	0.055^#^
Yes	81 (55%)	98 (67%)	
Weight loss (Kg)	2.70 ± 3.59	2.21 ± 3.47	0.237
ODI	17.9 ± 8.45	17.9 ± 8.45	0.95
JOA	18.0 ± 6.26	17.7 ± 6.08	0.684
VAS	7.34 ± 1.49	7.32 ± 1.37	0.903
ASIA	4.48 ± 0.896	4.52 ± 0.865	0.69
Occupation			
Non-farmer	64 (44%)	49 (34%)	0.093#
Farmer	82 (56%)	97 (66%)	
Race			
Non-Han	66 (45%)	76 (52%)	0.292
Han	80 (55%)	70 (48%)	
Days before surgery (days)	5.38 ± 5.80	4.82 ± 3.84	0.33
Smoking (pieces)	62.5 ± 190	62.8 ± 170	0.988
Drinking (ml)	645 ± 2,360	657 ± 2,680	0.968

# *This means that the P-value is <0.1*.

**Table 3:** Laboratory test results between complication and without complication groups after the propensity score matching analysis.

	**Without complications**	**Complications**	***P*** **-value**
	**(*N* = 146)**	**(*N* = 146)**	
Blood glucose (mmol/L)	5.23 ± 1.31	5.48 ± 1.95	0.19
Blood type			
A	26 (18%)	26 (18%)	0.992
B	44 (30%)	42 (29%)	
AB	11 (8%)	12 (8%)	
O	65 (45%)	66 (45%)	
C-reactive protein (mg/L)	0.370 ± 0.484	0.397 ± 0.491	0.632
Hepatitis B surface antigen			
Negative	135 (92%)	131 (90%)	0.538
Positive	11 (8%)	15 (10%)	
White blood cells (^*^10^9^/L)	7.33 ± 2.93	7.41 ± 2.87	0.823
Hemoglobin (g/L)	120 ± 17.2	118 ± 18.1	0.289
Platelets (^*^10^9^/L)	310 ± 94.8	309 ± 110	0.942
Percentage of neutrophils (%)	0.633 ± 0.111	0.645 ± 0.139	0.427
Percentage of lymphocytes (%)	0.229 ± 0.0896	0.219 ± 0.110	0.38
Absolute monocytes (^*^10^9^/L)	0.658 ± 0.312	0.668 ± 0.270	0.778
Percentage of monocytes (%)	0.0964 ± 0.0540	0.0943 ± 0.0313	0.684
Total bilirubin (umol/L)	8.89 ± 7.05	9.06 ± 9.71	0.862
Direct bilirubin (umol/L)	3.91 ± 4.57	4.06 ± 5.69	0.801
Indirect bilirubin (umol/L)	4.93 ± 3.51	4.99 ± 4.44	0.899
Total protein (g/L)	69.3 ± 7.76	69.8 ± 8.47	0.585
Albumin (g/L)	36.8 ± 5.45	36.6 ± 4.96	0.779
Aspartate aminotransferase (U/L)	25.1 ± 15.1	26.7 ± 24.1	0.519
Alanine aminotransferase (U/L)	23.9 ± 23.8	23.5 ± 23.5	0.91
AST/ALT	1.33 ± 0.668	1.50 ± 0.968	0.076^#^
Blood urea (mmol/L)	4.36 ± 1.79	5.30 ± 7.71	0.152
Blood creatinine (umol/L)	67.9 ± 18.6	71.5 ± 65.7	0.522
Blood uric acid (umol/L)	398 ± 191	382 ± 164	0.459
Erythrocyte sedimentation rate (mm/h)	43.9 ± 26.0	40.1 ± 23.9	0.197

# *This means that the P-value is <0.1*.

**Table 4:** Post-operative information comparison between complication and without complication groups after the propensity score matching analysis.

	**Without complications**	**Complications**	***P*** **-value**
	**(*N* = 146)**	**(*N* = 146)**	
Titanium cage			
No	72 (49%)	69 (47%)	0.815
Yes	74 (51%)	77 (53%)	
Internal fixation			
No	18 (12%)	19 (13%)	1
Yes	128 (88%)	127 (87%)	
Abscess			
No	17 (12%)	22 (15%)	0.491
Yes	129 (88%)	124 (85%)	
Albumin quantity (bottles)	0.740 ± 2.44	1.71 ± 2.82	0.002^**^
Recurrence			
No	141 (97%)	106 (73%)	<0.001^**^
Yes	5 (3%)	40 (27%)	
Surgical approach			
Anterior	97 (66%)	79 (54%)	0.086^#^
Posterior	44 (30%)	62 (42%)	
Anteroposterior	5 (3%)	5 (3%)	
Hospital stay (days)	11.7 ± 7.44	14.7 ± 9.34	0.003^**^
Transfusion			
No	99 (68%)	90 (62%)	0.327
Yes	47 (32%)	56 (38%)	
Local streptomycin			
No	22 (15%)	14 (10%)	0.213
Yes	124 (85%)	132 (90%)	
Operation time (minutes)	138 ± 60.5	162 ± 74.1	0.002^**^
Bleeding volume (ml)	476 ± 457	582 ± 1,070	0.269
Red blood cell transfusion (U)	1.16 ± 2.06	1.37 ± 2.78	0.455
Drainage (ml)	359 ± 287	419 ± 342	0.11
Kyphosis			
No	78 (53%)	70 (48%)	0.413
Yes	68 (47%)	76 (52%)	
Incision length (cm)	10.2 ± 3.56	11.2 ± 4.76	0.048^*^

# *This means that the P-value is <0.1*.

* *This means that the P-value is <0.05*.

** *This means that the P-value is <0.01*.

**Table 5:** The cost of hospitalization comparison between complication and without complication groups after the propensity score matching analysis.

	**Without complications**	**Complications**	***P*** **-value**
	**(*N* = 146)**	**(*N* = 146)**	
Antibacterial costs (RMB)	377 ± 1,110	1,220 ± 2,430	<0.001^**^
Western medicine costs (RMB)	4,510 ± 4,330	7,080 ± 7,650	<0.001^**^
Surgical costs (RMB)	6,220 ± 3,280	6,620 ± 3,920	0.34
Anesthesia costs (RMB)	782 ± 265	844 ± 367	0.099
Laboratory costs (RMB)	2,280 ± 1,360	2,900 ± 2,080	0.003^**^
Examine costs (RMB)	272 ± 422	425 ± 826	0.047^*^
Guardianship costs (RMB)	441 ± 186	791 ± 1,940	0.032^*^
Transfusion costs (RMB)	418 ± 676	680 ± 1,990	0.133
Oxygen costs (RMB)	103 ± 106	154 ± 210	0.009^**^
High consumable costs (RMB)	23,300 ± 16,500	26,400 ± 19,200	0.131
Care costs (RMB)	193 ± 141	273 ± 239	<0.001^**^
Consultation costs (RMB)	12.3 ± 29.3	29.3 ± 44.5	<0.001^**^
Total costs (RMB)	42,800 ± 24,200	51,700 ± 31,800	0.008^**^

* *This means that the P-value is <0.05*.

** *This means that the P-value is <0.01*.

We constructed a logistic regression model to further explore the risk factors that affected the complications. The parameters with *P*-values <0.1 were included in the logistic regression analysis, including appetite, occupation, AST/ALT, albumin quantity, surgical approach, operation time, and incision length. There was a significant difference in the albumin quantity using the logistic regression model analysis (Table 6). Furthermore, there was a significant difference in the intercept of the regression model (*P* < 0.05). However, there were no statistical differences in other parameters, including appetite, occupation, AST/ALT, surgical approach, operation time, and incision length (*P* > 0.05).

**Table 6:** Multivariate logistic regression analysis results.

	**β**	**OR**	**OR (95%CI)**	***P***
Intercept	−1.6262	0.1967	0.0437–0.8120	0.0281^*^
Albumin quantity	0.1570	1.1700	1.0273–1.3595	0.0265^*^
Operation time	0.0016	1.0016	0.9969–1.0064	0.5035
Incision length	0.0360	1.0367	0.9554–1.1288	0.3929
Appetite	0.2236	1.2506	0.6978–2.2480	0.4527
Surgical approach	0.2949	1.3430	0.74283–2.4528	0.3304
Occupation	0.2789	1.3216	0.7425–2.3577	0.3431
AST/ALT	0.1338	1.1432	0.8224–1.6285	0.4361

* *This means that the P-value is <0.05*.

**Figure 8:**
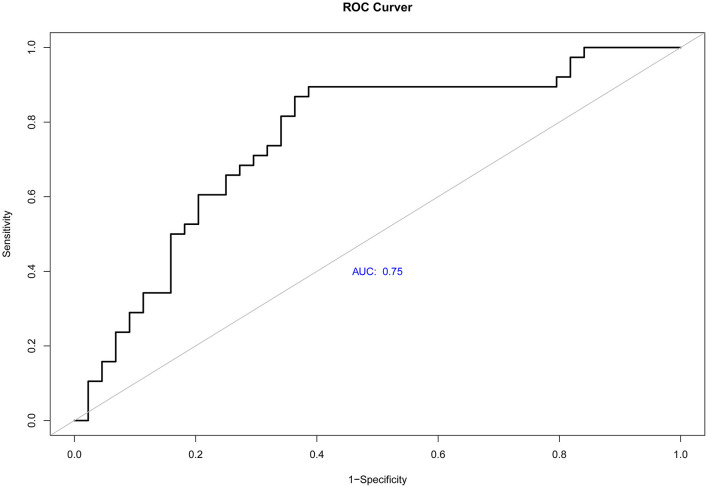
ROC curve. The area under the curve was 0.75.

The goodness of fit of the logistic regression model was assessed by HL-test and the chi-square value. *P*-values were 4.534 and 0.806, respectively. The confidence interval of the odds ratio (Table 6) was further used to evaluate the logistic regression model. The ROC curve was applied to evaluate the validity of the logistic regression model. The area under the curve (AUC) was 0.75 (Figure 8).

In this study, various complications occurred after spinal tuberculosis surgery during post-operative follow-up (Table 7). Eight patients were transferred to ICU for treatment due to post-operative respiratory failure, hypoxia, and inability to remove the ventilator. Wound infection occurred in 77 patients. After intensive wound management or two-stage debridement, they can recover well. Pedicle screw fracture occurred in 1 patient, requiring a second surgery to re-implant the internal fixation device.

**Table 7:** Various types of complications after spinal tuberculosis surgery.

**Complications**	**Numbers**
Transfer to ICU	8
Pulmonary embolism	2
Pneumonia	15
Intracranial infection	1
Pedicle screw fracture	1
Cerebrospinal fluid leak	2
Wound infection	77
Deep vein thrombosis of lower extremity	2
Pleural effusion	6
Other complications	32

## Discussion

Spinal tuberculosis surgery has become the most common treatment for spinal tuberculosis, including anterior (28), posterior (29), and combined anterior and posterior approaches (30). Moreover, the literature reports that spinal tuberculosis surgery includes minimally invasive procedures and combined open and minimally invasive procedures (7). However, post-operative complications are often unexpected and also a challenge for surgeons (31, 32). The complications of spinal tuberculosis vary depending on the location. The complications in cervical tuberculosis have been reported to be more than in non-cervical tuberculosis (33). In addition, post-operative complications of spinal tuberculosis vary depending on the surgical approach. The complications of the anterior approach were higher than in the posterior approach (7).

The parameters with a *P*-value <0.05 in the univariate analysis included albumin quantity, operation time, and incision length. Patients with classic spinal tuberculosis were often accompanied by weight loss (34); these patients who underwent surgical treatment were prone to poor wound healing due to post-operative nutritional insufficiency (35–37). Under such conditions, clinicians often use albumin supplementation to promote the recovery of patients (38). In addition, a longer surgical incision results in more secretion of fluid from the incision and increased susceptibility to wound infection (39). This could be the reason that the length of the incision in the complication group in this study was longer than that in the without complication group, and the albumin quantity in the former was more than that in the latter. If the operation time exceeded 3 h, the second group of antibiotics often needed to be increased, which potentially increased the risk of post-operative infection and other complications in case of prolonged operation time (40). Therefore, the operation time in the complication group was higher than in the without complication group.

Various costs, including hospital stay, laboratory costs, examination costs, guardianship costs, oxygen costs, and total costs, were higher in the complication group than in the without complication group. Research by Marah et al. showed that post-operative respiratory failure increased the cost of surgery by more than 50% (41). Mark et al. studied the relationship between surgical complications and hospitalization costs and found that hospitalization costs were on an average of $19,626 higher in patients with complications than in those without complications (42). The findings of Hunt et al. showed that post-operative complications not only increased the length of hospital stay but also increased the cost of hospitalization (43). This was consistent with the results of our study.

In the present study, only the albumin quantity was significantly different by multivariate logistic regression analysis. In addition, the albumin quantity in the complication group was higher than that in the without complication group. Li et al.'s study of spinal tuberculosis found that the albumin in the anterior approach group decreased from 37 g/L pre-operatively to 24.2 g/L post-operatively and that in the posterior approach group decreased from 38.6 to 28.2 g/L, with pneumonia and other complications in both groups of surgical approaches (44). A close relationship existed between albumin and post-operative complications (45), and albumin supplementation may accelerate the recovery of the post-operative condition (46).

The logistic regression model was tested through the HL test method, and the *P*-value was 0.806 (*P* > 0.05), which implied no difference between the logistic regression model and the actual application. The value of the area under the ROC curve was 0.75, implying that this regression model can be used in clinical applications.

PSM analysis strategies, such as tumor chemotherapy (47), cervical spine surgery (48), robot-assisted surgery (49), and other areas, are widely used in medical research. The PSM analysis can reduce the difference between the two groups of comparable data and decrease the effect of interfering factors on the results by adjusting the balance of baseline data (22). Such a model allows the researchers to measure the probability of an outcome event occurring between treatment and control groups (50). The pre-matched imbalance and post-matched balance were assessed using the balance standardized differences (23). We found a significant difference in the laboratory results of albumin value between the complication and without complication groups, which inevitably became a disturbing factor for another result that the albumin quantity was an independent risk by the logistic regression analysis. The albumin values of the two groups before the operation were adjusted to be the same by the PSM analysis, and the results of the study were more comparable.

However, this study had certain limitations. (1) Some data were lost after the PSM analysis. (2) More sample data should be collected to obtain more pairs. (3) This subject was a single-center study, and the results need to be verified by a multi-center study.

## Conclusion

In this study, the costs in the complication group were generally higher than in the without complication group. Multivariate logistic regression analysis found that albumin quantity could be an independent factor for predicting post-operative complications of spinal tuberculosis. Therefore, surgeons should pay more attention to the patient's economy and the occurrence of post-operative complications in clinical work.

## Data Availability Statement

The original contributions presented in the study are included in the article/supplementary material, further inquiries can be directed to the corresponding author/s.

## Ethics Statement

The studies involving human participants were reviewed and approved by Ethics Committee of the First Affiliated Hospital of Guangxi Medical University. The patients/participants provided their written informed consent to participate in this study.

## Author Contributions

LC wrote the article and prepared Figures 1–8 and Tables 1–6. All authors reviewed the article, including SH, TC, HL, TL, ZY, WC, XS, JJ, JC, HG, YY, SL, CY, BF, SW, and XZ. All authors contributed to the article and approved the submitted version.

## Funding

This work was sponsored by the National Natural Science Foundation of China (81560359) and National Natural Science Foundation of China (81860393). Funding bodies had no role in the study design, collection, analysis, and interpretation of the data or in writing the manuscript.

## Conflict of Interest

The authors declare that the research was conducted in the absence of any commercial or financial relationships that could be construed as a potential conflict of interest.

## Publisher's Note

All claims expressed in this article are solely those of the authors and do not necessarily represent those of their affiliated organizations, or those of the publisher, the editors and the reviewers. Any product that may be evaluated in this article, or claim that may be made by its manufacturer, is not guaranteed or endorsed by the publisher.
